# Prevalence of and Factors Associated With Patient Nondisclosure of Medically Relevant Information to Clinicians

**DOI:** 10.1001/jamanetworkopen.2018.5293

**Published:** 2018-11-30

**Authors:** Andrea Gurmankin Levy, Aaron M. Scherer, Brian J. Zikmund-Fisher, Knoll Larkin, Geoffrey D. Barnes, Angela Fagerlin

**Affiliations:** 1 Department of Social Sciences, Middlesex Community College, Middletown, Connecticut; 2 Department of Internal Medicine, University of Iowa, Iowa City; 3 School of Public Health, University of Michigan, Ann Arbor; 4 Department of Oncology, School of Medicine, Wayne State University, Detroit, Michigan; 5 Cardiovascular Medicine and Vascular Medicine, University of Michigan Health System, Ann Arbor; 6 Department of Population Health Sciences, University of Utah, Salt Lake City

## Abstract

**Importance:**

Patient failure to disclose medically relevant information to clinicians can undermine patient care or even lead to patient harm.

**Objective:**

To examine the frequency of patients failing to disclose to their clinicians information that is relevant to their care and their reasons for doing so.

**Design, Setting, and Participants:**

Two national nonprobability samples were recruited to participate in an online survey, one using Amazon’s Mechanical Turk (MTurk) from March 16 to 30, 2015 (2096 respondents), followed by one using Survey Sampling International (SSI) from November 6 to 17, 2015 (3011 respondents). Data analysis was conducted from September 28 to October 8, 2018. After dropping respondents meeting the exclusion criteria, the final sample sizes were 2011 (MTurk) and 2499 (SSI).

**Main Outcomes and Measures:**

The primary outcome measures were self-reported nondisclosure of 7 types of information to their clinician (eg, did not understand instructions, medication use) and reasons for nondisclosure (eg, embarrassment, not wanting to be judged).

**Results:**

There was a total of 4510 overall respondents. Of 2096 respondents, 2013 completed the MTurk survey (96.0% completion rate) and 2011 were included in the analysis. Of 3011 respondents, 2685 completed the SSI survey (89.2% completion rate) and 2499 were included in the analysis. The mean (SD) age of the participants was 36 (12.4) years for MTurk and 61 (7.59) years for SSI. Both samples were predominantly white (MTurk: 1696 [84.3%]; SSI: 1968 [78.8%]). A total of 1630 MTurk participants (81.1%) and 1535 SSI participants (61.4%) avoided disclosing at least 1 type of information. Disagreeing with the clinician’s recommendation (MTurk: 918 of 2010 respondents [45.7%]; SSI: 785 of 2497 respondents [31.4%]) and not understanding the clinician’s instructions (MTurk: 638 of 2009 respondents [31.8%]; SSI: 607 of 2497 respondents [24.3%]) were the most common occurrences. The most commonly reported reasons for nondisclosure included not wanting to be judged or lectured (MTurk: 81.8% [95% CI, 79.8%-83.9%]; SSI: 64.1% [95% CI, 61.5%-66.7%]), not wanting to hear how harmful the behavior is (MTurk: 75.7% [95% CI, 73.5%-78.0%]; SSI: 61.1% [95% CI, 58.5%-63.8%]), and being embarrassed (MTurk: 60.9% [95% CI, 58.9%-62.9%]; SSI: 49.9% [95% CI, 47.8%-52.1%]). In both samples, participants who were women (MTurk: odds ratio [OR], 1.88 [95% CI, 1.49-2.37]; SSI: OR, 1.38 [95% CI, 1.17-1.64]), younger (MTurk: OR, 0.98 [95% CI, 0.97-0.99]; SSI: OR, 0.98 [95% CI, 0.97-0.99]), and with worse self-rated health (MTurk: OR, 0.87 [95% CI, 0.76-0.99]; SSI: OR, 0.80 [95% CI, 0.72-0.88]) were more likely to report withholding information.

**Conclusions and Relevance:**

Many respondents in these surveys intentionally withhold important information from their clinicians and were most likely to do so when they disagreed with or misunderstood their clinician’s instructions. A better understanding of how to increase patients’ comfort with reporting this information may improve the clinician-patient relationship and patient care.

## Key Points

**Question:**

What medically relevant information do patients withhold from their clinicians, and why do they do so?

**Findings:**

In 2 national, nonprobability online surveys of 4510 US adults, most participants reported withholding at least 1 of 7 types of medically relevant information, especially when they disagreed with the clinician’s recommendations or misunderstood the clinician’s instructions. The most commonly reported reasons for not disclosing information included not wanting to be judged or hear how harmful their behavior is.

**Meaning:**

Patients commonly withhold medically relevant information from their clinicians, a pattern that likely inhibits the quality of patient care.

## Teaser

This survey study evaluates adult patients’ frequency of and reasons for withholding information from health care professionals that may affect diagnoses and therapy.

## Introduction

Patient disclosure to clinicians is an essential element of the clinician-patient relationship and patient care. Clinicians rely on patients to disclose their symptoms, health behaviors, and thoughts and feelings so that clinicians can make appropriate diagnoses and treatment recommendations. Without accurate information, clinician recommendations and decisions may even harm patients. For example, if a patient withholds details about their current medication use (including over-the-counter products), a clinician might unknowingly prescribe a contraindicated medication.

Despite the importance of patients providing accurate information to clinicians, there is a long-standing conventional belief in the field of medicine that patients lie to clinicians.^1^ For example, it is said that clinicians need to double their patients’ reports about alcohol use and halve the reported amount of exercise to gain a more accurate picture. This conventional belief suggests that patients might not be providing clinicians with essential medical information.

The limited research conducted to date suggests that, consistent with this conventional belief, a substantial proportion of patients withhold important information from their clinicians. For instance, the Health Information National Trends Survey revealed that 12.3% of patients reported ever withholding information from a clinician out of concern for the security or privacy of their medical records.^2^

Other studies have examined this question, but the scope was limited, such as only examining one issue (eg, alcohol use, use of complementary and alternative medicine).^3,4,5,6,7,8,9,10^ For example, one review article summarized the results of studies on the nondisclosure of complementary and alternative medicine use among patients with cancer, finding nondisclosure rates ranging from 20% to 77%. Of the studies in this review article that assessed reasons for the nondisclosure, clinicians not asking; patient anticipation of clinician disapproval, disinterest, or inability to help; and patient perceptions that information about their complementary and alternative medicine use is irrelevant to their traditional care were the most commonly reported.^8^ In a study of nondisclosure about depression, the most commonly reported reasons for nondisclosure were medication aversion, that treating depression is not the clinician’s job, and concern about the information being in their medical record.^10^ To our knowledge, however, no study has more extensively explored which types of information patients commonly withhold from clinicians and their stated reasons for doing so.

Thus, in this research, we examined what information patients are most likely to avoid telling a clinician, with a focus on types of information that are basic and essential to health care (eg, medication use, health behaviors, disagreement with recommendations, or lack of understanding of instructions). We also examined the characteristics that are associated with withholding information from clinicians and the reasons for this nondisclosure, distinguishing between, for example, nondisclosure due to embarrassment vs privacy reasons.

## Methods

### Participants

We conducted 2 distinct surveys to explore patient nondisclosure to clinicians. The study was reviewed by the institutional review board of the University of Michigan Medical School. The first survey, Amazon’s Mechanical Turk (MTurk), was approved, and the second, Survey Sampling International (SSI), was deemed exempt. Consent to participate was indicated electronically immediately before the user was routed to the webpage with the start of the survey. The participants were provided with financial compensation from MTurk and reward points from SSI. This study followed the American Association for Public Opinion Research (AAPOR) reporting guideline.

From March 16 to 30, 2015, we recruited a nonprobability sample of US adults aged 18 and older from internet users who participate in the MTurk marketplace. MTurk is a population of internet users who participate in tasks (eg, surveys) in exchange for small monetary compensation and has been shown to be a good source of reliable data,^11^ but the sample population is younger than the US population.

To complement these data, we recruited a second nonprobability sample of older US adults (aged ≥50 years) using SSI, a demographically diverse web panel, from November 6 to 17, 2015. Quotas were set for sex, age, and race/ethnicity to match the distribution of those characteristics in the US population. Sample sizes were determined to estimate the prevalence of any particular behavior within 2 percentage points. The final analysis was conducted from September 28 to October 8, 2018.

### Survey Instrument

The survey instrument was developed with input from a team of physicians, health services researchers, and psychologists, as well as through interviews, group meetings, and pilot testing with the lay public. The survey assessed a number of common contexts in which a patient may fail to disclose relevant information to a clinician. Specifically, it asked whether participants had “ever avoided telling a health care provider” that they (1) did not understand the provider’s instructions, (2) disagreed with the provider’s recommendation, (3) did not exercise or did not exercise regularly, (4) had an unhealthy diet or how unhealthy their diet was, (5) took a certain medication (ie, deliberately did not mention a certain agent), (6) did not take their prescription medication as instructed, and (7) took someone else’s prescription medication. The survey defined “health care provider” as “any medical care giver, such as a doctor, physician's assistant, or nurse.”

Participants sequentially responded to each of the 7 questions. If they reported that they had ever avoided telling a clinician that particular piece of information, they were routed to a page that assessed their reasons for the nondisclosure of that information before moving on to the next disclosure item. These reasons were consistent across similar items but different where appropriate. For instance, all items asking about a health behavior (eg, having an unhealthy diet) included, “I didn’t want to be judged or lectured about my behavior” as a reason for the nondisclosure, but this reason was omitted for “disagreed with the provider’s recommendation” and “did not understand the provider’s instructions.” Participants could choose all the reasons that applied to them.

Participants also reported their sex (0, male; 1, female), age, race/ethnicity (by indicating whether they are Hispanic or Latino/Latina and by selecting which of the following applied to them: white or Caucasian, black or African American, American Indian or Alaskan Native, Asian or Asian American, Pacific Islander or Native Hawaiian, or other), self-reported general health (1, excellent; 2, very good; 3, good; 4, fair; and 5, poor), and whether they had any chronic health conditions.

### Patient Involvement

Members of the lay public, all of whom have been a patient at some point, assisted with the development of the survey through discussions, interviews, and pilot testing. The survey was refined where omissions or lack of clarity were identified through the pilot testing, and additional pilot testing was done to ensure that the online survey worked properly.

### Statistical Analysis

We recoded race and ethnicity into a dichotomous variable, with participants selecting white being coded as 1 and participants not selecting white being coded as 0. Health was reverse coded so that higher values indicate better self-reported health. To assess how often a reason was given, an aggregate measure for each reason was created by dividing the number of times a reason was selected by the total number of nondisclosures (eg, indicating “embarrassed” for 2 of the 3 types of information that a respondent reported failing to disclose would yield a value of 66.7%).

We report descriptive statistics, the percentage of participants reporting ever having avoided telling a clinician each of the 7 types of medically relevant information, and the reasons they chose for doing so. We used multiple logistic regression to examine the demographic characteristics that were associated with whether the participant reported avoiding telling a clinician any of the 7 types of information. A statistical significance level of *P* < .05 was used. All analyses were conducted using SPSS, version 22 (SPSS Inc) or Stata, version Stata SE 14 (StataCorp).

## Results

Once the survey was initiated, 2013 of 2096 MTurk participants (96.0%) and 2685 of 3011 SSI participants (89.2%) completed it. Two participants were excluded from the MTurk sample because they asked that their data not be used in analyses, and 186 participants were excluded from the SSI sample because they reported ages outside of the 50- to 100-year required age range. Thus, there was a total of 4510 participants. MTurk participants (n = 2011) had a mean (SD) age of 36 (12.4) years (range, 18-79 years), 974 of 2006 (48.6%) had completed at least a bachelor’s degree, 1696 (84.3%) identified as white, and 1210 of 1992 (60.7%) identified as female. A total of 1706 (84.8%) participants rated their health as good, very good, or excellent, and 450 of 2003 (22.5%) reported having a chronic illness.

Participants in the SSI sample (n = 2499) had a mean (SD) age of 61 (7.59) years (range, 50-91 years), 1023 of 2484 (41.2%) had completed a bachelor’s degree or more, 1968 of 2499 (78.8%) identified as white, and 1273 of 2491 (51.1%) identified as female. A total of 1972 of 2480 (79.5%) participants rated their health as good, very good, or excellent, and 969 of 2473 (39.2%) reported having a chronic illness.

### Patient Nondisclosure to Clinicians

In the MTurk sample, 1630 of 2011 participants (81.1%) reported having ever avoided telling a clinician any of the 7 types of medically relevant information. The SSI sample had similar rates of nondisclosure; 1535 of 2499 participants (61.4%) reported having ever avoided telling a clinician any of the 7 types of medically relevant information.

Table 1 presents the frequency and percentage of participants in both samples who reported that they ever avoided telling a clinician each of the 7 types of information. Failures to disclose were highest for items related to clinician communication. Disagreeing with the clinician’s recommendation (MTurk: 918 of 2010 respondents [45.7%]; SSI: 785 of 2497 respondents [31.4%]) and not understanding the clinician’s instructions (MTurk: 638 of 2009 respondents [31.8%]; SSI: 607 of 2497 respondents [24.3%]) were the most common occurrences, followed by not disclosing relevant health behaviors, such as an unhealthy diet (MTurk: 493 of 2009 respondents [24.5%]; SSI: 506 of 2497 respondents [20.3%%]) and not exercising (MTurk: 446 of 2008 respondents [22.2%]; SSI: 538 of 2495 respondents [21.6%]). While nondisclosure about various types of information about medication use was less common compared with the other types of information, a substantial minority of respondents (8.8%-22.5%) indicated nondisclosure of this type of information to their clinician.

**Table 1.** Percentage of Participants Who Avoided Telling a Clinician Each Type of Information

Type of Information	Ever Avoided Informing the Clinician, No. (%)	
	MTurk (n = 2011)	SSI (n = 2499)
Disagreed with clinician’s recommendation	918 (45.7) (n = 2010)	785 (31.4) (n = 2497)
Did not understand clinician’s instructions	638 (31.8) (n = 2009)	607 (24.3) (n = 2497)
Had unhealthy diet	493 (24.5) (n = 2009)	506 (20.3) (n = 2491)
Did not take prescription medication as instructed	453 (22.5) (n = 2011)	439 (17.6) (n = 2491)
Did not exercise	446 (22.2) (n = 2008)	538 (21.6) (n = 2495)
Took a certain medication (deliberately did not mention a certain medication)	311 (15.5) (n = 2009)	258 (10.4) (n = 2489)
Took someone else’s prescription medication	280 (13.9) (n = 2009)	219 (8.8) (n = 2491)

Abbreviations: MTurk, Amazon’s Mechanical Turk; SSI, Survey Sampling International.

### Reasons for Nondisclosure

Table 2 reports the percentage of times a reason for nondisclosure was indicated for both MTurk and SSI participants. The 5 most commonly indicated reasons for participants not disclosing information to their clinician were not wanting to be judged or lectured (MTurk: 81.8% [95% CI, 79.8%-83.9%]; SSI: 64.1% [95% CI, 61.5%-66.7%]), not wanting to hear how harmful the behavior is (MTurk: 75.7% [95% CI, 73.5%-78.0%]; SSI: 61.1% [95% CI, 58.5%-63.8%]), embarrassment (MTurk: 60.9% [95% CI, 58.9%-62.9%]; SSI: 49.9% [95% CI, 47.8%-52.1%]), not wanting the clinician to think that they are a difficult patient (MTurk: 50.8% [95% CI, 48.7%-52.9]; SSI: 38.1% [95% CI, 36.0%-40.3%]), and not wanting to take up more of the clinician’s time (MTurk: 45.2% [95% CI, 42.6%-47.9%]; SSI: 35.9% [95% CI, 33.2%-38.7%]). All explanations that were offered for each 33.2%-item are provided in eTable 1 and eTable 2 in the Supplement.

**Table 2.** Percentage of Times a Reason Was Selected for Avoiding Telling the Clinician Collapsed Across Types of Information^a^

Reason	% (95% CI)	
	MTurk	SSI
I didn’t want to be judged or get a lecture about my behavior.	81.8 (79.8-83.9)	64.1 (61.5-66.7)
I didn’t want to hear how bad [insert behavior] is for me.	75.7 (73.5-78.0)	61.1 (58.5-63.8)
I was embarrassed to admit that I [insert item].	60.9 (58.9-62.9)	49.9 (47.8-52.1)
I didn’t want the health care provider to think that I’m a difficult patient.	50.8 (48.7-52.9)	38.1 (36.0-40.3)
I didn’t want to take up any more of the health care provider’s time.	45.2 (42.6-47.9)	35.9 (33.2-38.7)
I didn’t think it mattered.	38.6 (36.6-40.6)	32.9 (30.9-35.0)
I didn’t want the health care provider to think that I’m stupid.	37.6 (35.7-39.6)	30.6 (28.6-32.7)
I didn’t want this information in my medical record.	34.5 (32.0-37.0)	30.6 (28.1-33.1)
I didn’t want to have to make a difficult change (ie, [insert behavior change]) that the health care provider would then recommend.	32.7 (30.3-35.2)	35.2 (32.7-37.7)
I didn’t think the health care provider could help me with this problem.	27.7 (25.2-30.3)	28.9 (26.2-31.6)
I wanted the health care provider to like me.	20.6 (18.8-22.4)	16.0 (14.4-17.7)
I had a bad experience with telling a health care provider that I didn't understand their instructions.	15.6 (14.1-17.1)	17.3 (15.7-18.9)
I didn’t want the health care provider to tell someone in my family.	12.7 (10.9-14.4)	13.3 (11.5-15.2)

Abbreviations: MTurk, Amazon’s Mechanical Turk; SSI, Survey Sampling International.

^a^ Denominator was the total number of information types that a person failed to disclose to the clinician. Column totals may exceed 100% because participants could check multiple reasons.

### Risk Factors Associated With Nondisclosure to Clinicians

Participants in the MTurk sample who were female (odds ratio [OR], 1.88; 95% CI, 1.49-2.37), younger (OR, 0.98; 95% CI, 0.97-0.99), white (OR, 1.49; 95% CI, 1.11-2.02), or had worse self-rated health (OR, 0.87; 95% CI, 0.76-0.99) or chronic illness (OR, 1.40; 95% CI, 1.02-1.14) were significantly more likely to have ever avoided sharing at least 1 of the 7 types of information with their clinician.

The SSI results were similar to the MTurk results. Participants who were female (OR, 1.38; 95% CI, 1.17-1.64), younger (OR, 0.98; 95% CI, 0.97-0.99), or had worse self-rated health (OR, 0.80; 95% CI, 0.72-0.88) were significantly more likely to have ever avoided sharing at least 1 of the 7 types of information with their clinician. Race and having a chronic illness were not significantly associated with nondisclosure behavior in the SSI sample, and educational level was not significantly associated with nondisclosure in either sample.

## Discussion

The results of this research reveal that most patients have withheld medically relevant information from their clinician at least once, especially regarding disagreement with clinician recommendations and failure to understand clinician’s instructions, and that the most commonly reported reasons for doing so were that patients did not want to be negatively judged by their clinician, did not want to hear how harmful the behavior in question was, or were embarrassed. These findings indicate that clinicians at times do not receive accurate, relevant information from patients, which may affect clinicians’ diagnoses and recommendations as well as patient care.

Survey respondents who were sicker (ie, worse self-reported health, had chronic conditions) were slightly, but significantly, more likely to withhold information from their clinician, indicating that the very patients who are in greatest need of high-quality health care because of the complexity of their health may be more likely to compromise their care by withholding important information from their clinician.

These results are consistent with the long-standing conventional belief that patients lie to their clinicians and with the studies that have examined this question for single issues (eg, alternative medicine use) or in general.^1,2,3,4,5,6,7,8,9^ Unlike the previous studies in this area that have focused on a particular issue (eg, alcohol use, use of complementary and alternative medicine), to our knowledge, this is the first study to more broadly examine patients’ nondisclosure of medically relevant information to their clinician for a variety of common experiences that patients may have and their reasons for withholding this information. Our study highlights that nondisclosure can occur for even relatively mundane interactions with a clinician (eg, not understanding their instructions) despite the potential consequences of these nondisclosures.

Although we chose our types of information based on common scenarios that patients may face, future research in this area could more systematically examine how characteristics, such as the potential severity of the consequences of nondisclosure or the level of stigmatization of a health behavior, affect nondisclosure rates. In addition, researchers could examine the frequency of nondisclosure for different types of medically relevant information, although this work would be more challenging to complete given that people are likely to be less accurate about how many times they have failed to disclose information to their clinician as opposed to whether they ever have or have not failed to disclose information.

### Limitations

The current research is not without limitations. First, both studies used online samples. Although online samples are not fully representative, they allowed for a larger sample size than data collection completed in a clinical setting and ensured that we included older adults and a demographically diverse sample. Second, in a study examining the extent to which patients are not truthful with their clinicians, we acknowledge the likelihood that participants may have not been entirely truthful in their survey responses. However, we expect that, if participants were not entirely forthcoming, they provided answers that were more socially desirable (ie, minimizing the extent to which they withhold information from clinicians), thereby making our results an underestimate of the extent to which patients withhold information from their clinicians. Third, while the study captured the percentage of people who avoid disclosing information to clinicians, it cannot speak to the extent of the nondisclosure (eg, whether patients who are not taking a prescription medication at all state that they were taking it as instructed or merely that they take it less than instructed), frequency of the nondisclosure, or other types of information that are withheld beyond what was included in our study. Fourth, as noted, we tailored the reasons for withholding information to the item that participants were explaining, but there were some inconsistencies in this regard. For instance, we included, “I didn’t think the health care provider could help me with this problem,” as an explanation that participants could select for the unhealthy diet item but not for the no-exercise item. Although the presence or absence of this explanation may have affected other explanations that participants selected, the results regarding participants’ reasons for withholding information are still informative. Fifth, because we did not randomize the order of presentation of the items, an order association is possible. It is also possible that these reasons that participants selected may be post hoc explanations rather than original determinants of the nondisclosure.

## Conclusions

Despite these limitations, the results of this research highlight an important and concerning reality in health care. If patients are withholding information from clinicians as frequently as this research suggests, then clinicians are routinely not receiving the information that they need to provide high-quality care to patients, especially sicker patients. The clinician-patient relationship requires honest and open communication between both parties to maximize the therapeutic benefit and avoid potential harms. Future research should test interventions aiming to increase the trust and communication between patients and their clinicians as well as patients’ comfort with disclosing information to their clinicians.

## Notes

Supplement. **eTable 1.** Frequency With Which Amazon’s Mechanical Turk (MTurk) Participants Chose Each Reason for Avoiding Telling Clinician Each Type of Information **eTable 2.** Frequency With Which Survey Sampling International (SSI) Participants Chose Each Reason for Avoiding Telling Clinician Each Type of Information
