# Supplementary material for: Prevalence of and Factors Associated With Patient Nondisclosure of Medically Relevant Information to Clinicians
Source: JAMA Netw Open. 2018 Nov 30;1(7):e185293. doi: 10.1001/jamanetworkopen.2018.5293 (PMC6324389; doi:10.1001/jamanetworkopen.2018.5293)
Supplement: Supplement. — eTable 1. Frequency With Which Amazon’s Mechanical Turk (MTurk) Participants Chose Each Reason for Avoiding Telling Clinician Each Type of Information eTable 2. Frequency With Which Survey Sampling International (SSI) Participants Chose Each Reason for Avoiding Telling Clinician Each Type of Information [file jamanetwopen-1-e185293-s001.pdf]

Supplementary Online Content

Levy AG, Scherer AM, Zikmund-Fisher BJ, Larkin K, Barnes GD, Fagerlin A. Prevalence of and factors associated with patient nondisclosure of medically relevant information to clinicians. *JAMA Netw Open*. 2018;1(7):e185293. doi:10.1001/jamanetworkopen.2018.5293

eTable 1. Frequency With Which Amazon's Mechanical Turk (MTurk) Participants Chose Each Reason for Avoiding Telling Clinician Each Type of Information
eTable 2. Frequency With Which Survey Sampling International (SSI) Participants Chose Each Reason for Avoiding Telling Clinician Each Type of Information

This supplementary material has been provided by the authors to give readers additional information about their work.

© 2018 Levy AG et al. *JAMA Network Open.*

eTable 1. Frequency With Which Amazon's Mechanical Turk (Mturk) Participants Chose Each Reason for Avoiding Telling Clinician Each Type of Information

	**Information participant avoided telling**						
Reason	**Didn't understand provider's instructions**	**Disagreed with provider's recommendation**	**Didn't exercise**	**Had unhealthy diet**	**Took a certain medication**	**Did not take prescription medication as instructed**	**Took someone else's prescription medication**
I was embarrassed to admit that I [insert item].	523/638 (82.0%)	302/918 (32.9%)	399/446 (89.5 %)	438/493 (88.8%)	155/311 (49.8%)	271/453 (59.8%)	163/280 (58.2%)
I didn't want to be judged or get a lecture about my behavior.	-	-	389/446 (87.2 %)	440/493 (89.3%)	216/311 (69.5%)	373/453 (82.3%)	242/280 (86.4%)
I didn't want to hear how bad [insert behavior] is for me.	-	-	347/446 (77.8 %)	414/493 (84.0%)	199/311 (64.0%)	352/453 (77.7%)	242/280 (86.4%)
I didn't want the health care provider to think that I'm stupid.	479/638 (75.1%)	318/918 (34.6%)	135/446 (30.3 %)	184/493 (37.3%)	79/311 (25.4%)	167/453 (36.9%)	74/280 (26.4%)
I didn't want the health care provider to think that I'm a difficult patient.	422/638 (66.1%)	656/918 (71.5%)	128/446 (28.7 %)	151/493 (30.6%)	-	266/453 (58.7%)	84/280 (30.0%)
I didn't want this information in my medical record.	-	-	128/446 (28.7 %)	150/493 (30.4%)	184/311 (59.2%)	129/453 (28.5%)	171/280 (61.1%)

© 2018 Levy AG et al. *JAMA Network Open.*

I didn't want to take up any more of the health care provider's time.	376/638 (58.9%)	349/918 (38.0%)	-	-	-	-	-
I didn't think it mattered.	291/638 (45.6%)	436/918 (47.5%)	103/446 (23.1%)	115/493 (23.3%)	144/311 (46.3%)	143/453 (31.6%)	106/280 (37.9%)
I didn't want to have to make a difficult change (i.e. [insert behavior change]) that the health care provider would then recommend.	-	-	168/446 (37.7%)	237/493 (48.1%)	112/311 (36.0%)	134/453 (29.6%)	74/280 (26.4%)
I didn't think the health care provider could help me with this problem.	-	-	-	165/493 (33.5%)	82/311 (26.4%)	132/453 (29.1%)	63/280 (22.5%)
I wanted the health care provider to like me.	182/638 (28.5%)	216/918 (23.5%)	113/446 (25.3%)	121/493 (24.5%)	61/311 (19.6%)	103/453 (22.7%)	60/280 (21.4%)
I had a bad previous experience with telling a health care provider [insert information].	131/638 (20.5%)	236/918 (25.7%)	55/446 (12.3%)	58/493 (11.8%)	48/311 (15.4%)	61/453 (13.5%)	26/280 (9.3%)
I didn't want the health care provider to tell someone in my family.	-	-	41/446 (9.2%)	68/493 (13.8%)	74/311 (23.8%)	59/453 (13.0%)	56/280 (20.0%)

- = this reason was not offered as an option for this item
Column totals exceed 100% because participants could check all that apply.
N's represent the # of participants who reported having avoided telling a clinician that type of information

eTable 2. Frequency With Which Survey Sampling International (SSI) Participants Chose Each Reason for Avoiding Telling Clinician Each Type of Information

	**Information participant avoided telling**						
Reason	**Didn't understand provider's instructions**	**Disagreed with provider's recommendation**	**Didn't exercise**	**Had unhealthy diet**	**Took a certain medication**	**Did not take prescription medication as instructed**	**Took someone else's prescription medication**
I was embarrassed to admit that I [insert item].	415/607 (68.4%)	232/785 (29.6%)	409/538 (76.0%)	365/506 (72.1%)	100/258 (38.8%)	239/439 (54.4%)	121/219 (55.3%)
I didn't want to be judged or get a lecture about my behavior.	-	-	362/538 (67.3%)	364/506 (71.9%)	169/258 (65.5%)	284/439 (64.7%)	148/219 (67.6%)
I didn't want to hear how bad [insert behavior] is for me.	-	-	345/538 (64.1%)	346/506 (68.4%)	153/258 (59.3%)	284/439 (64.7%)	162/219 (74.0%)
I didn't want the health care provider to think that I'm stupid.	361/607 (59.5%)	233/785 (29.7%)	150/538 (27.9%)	181/506 (35.8%)	75/258 (29.1%)	138/439 (31.4%)	73/219 (33.3%)
I didn't want the health care provider to think that I'm a difficult patient.	305/607 (50.3%)	399/785 (50.8%)	201/538 (37.4%)	192/506 (37.9%)	85/258 (33.0%)	201/439 (45.8%)	64/219 (29.2%)
I didn't want this information in my medical record.	-	-	154/538 (28.6%)	169/506 (33.4%)	113/258 (43.8%)	135/439 (30.8%)	110/219 (50.2%)

© 2018 Levy AG et al. *JAMA Network Open.*

I didn't want to take up any more of the health care provider's time.	287/607 (47.3%)	244/785 (31.1%)	-	-	-	-	-
I didn't think it mattered.	219/607 (36.1%)	308/785 (39.2%)	149/538 (27.7%)	137/506 (27.1%)	113/258 (43.8%)	144/439 (32.8%)	87/219 (39.7%)
I didn't want to have to make a difficult change (i.e. [insert behavior change]) that the health care provider would then recommend.	-	-	210/538 (39.0%)	252/506 (49.8%)	123/258 (47.7%)	133/439 (30.3%)	52/219 (23.74%)
I didn't think the health care provider could help me with this problem.	-	-	-	188/506 (37.2%)	90/258 (34.9%)	127/439 (28.9%)	52/219 (23.7%)
I wanted the health care provider to like me.	134/607 (22.1%)	149/785 (19.0%)	104/538 (19.3%)	111/506 (21.9%)	49/258 (19.0%)	88/439 (20.1%)	44/219 (20.1%)
I had a bad previous experience with telling a health care provider [insert information].	172/607 (28.3%)	222/785 (28.3%)	84/538 (15.6%)	96/506 (19.0%)	60/258 (23.3%)	84/439 (19.1%)	41/219 (18.7%)
I didn't want the health care provider to tell someone in my family.	-	-	78/538 (14.5%)	84/506 (16.6%)	46/258 (17.8%)	73/439 (16.6%)	41/219 (18.7%)

- = this reason was not offered as an option for this item
Column totals exceed 100% because participants could check all that apply.
N's represent the # of participants who reported having avoided telling a clinician that type of information

© 2018 Levy AG et al. *JAMA Network Open.*
